# IGFBP3 induces PD-L1 expression to promote glioblastoma immune evasion

**DOI:** 10.1186/s12935-024-03234-3

**Published:** 2024-02-07

**Authors:** Leilei Zhao, Yudi Wang, Peizheng Mu, Xuehua Zhang, Ruomei Qi, Yurui Zhang, He Zhang, Xiao Zhu, Zhouyan Dong, Yucui Dong

**Affiliations:** 1 Department of Immunology, Binzhou Medical University, Guanhai Road 346, Yantai, 264003 Shandong China; 2 School of Computer and Normal Engineering, Yantai University, Qingquan Road 30, Yantai, 264005 Shandong China; 3 Department of Precision Biomedical Laboratory, Liaocheng People’s Hospital, Liaocheng, Shandong China; 4 Department of Immunology, Qiqihar Medical University, Qiqihar, Heilongjiang China; 5 Department of Pathogenic Biology, Binzhou Medical University, Guanhai Road 346, Yantai, 264003 Shandong China

**Keywords:** Glioblastoma, IGFBP3, PD-L1, JAK2, STAT3, Target therapy

## Abstract

**Background:**

Glioblastoma (GBM) characterized by immune escape is the most malignant primary brain tumors, which has strong immunosuppressive effect. Programmed death ligand-1 (PD-L1) is a recognized immunosuppressive member on the surface of tumor cells, and plays a crucial role in immune evasion of tumors. Actually, little is known about the regulation of PD-L1 expression in GBM. Insulin-like growth factor binding protein 3 (IGFBP3) is upregulated in GBM and is related to poor patient prognosis. However, it remains unclear whether IGFBP3 plays a role in the regulation of PD-L1 expression in GBM.

**Methods:**

The role of IGFBP3 in the glioma immune microenvironment was investigated using the CIBERSORT algorithm. The correlation between IGFBP3 and PD-L1 expression was analyzed using TCGA and CGGA databases. QRT-PCR, immunoblotting and RNA-seq were used to examine the regulatory effect of IGFBP3 on PD-L1 expression. Co-culture assay, cell counting kit (CCK-8), qRT-PCR, ELISA and flow cytometry were performed to explore the function of IGFBP3 in inducing immunosuppression. The biological role of IGFBP3 was verified using immunohistochemical, immunofluorescence and mice orthotopic tumor model.

**Results:**

In this study, we analyzed immune cells infiltration in gliomas and found that IGFBP3 may be associated with an immunosuppressive microenvironment. Then, by analyzing TCGA and CGGA databases, our results showed that IGFBP3 and PD-L1 expression were positively correlated in GBM patients, but not in LGG patients. In vitro experiments conducted on different GBM cell lines revealed that the overexpression of IGFBP3 led to an increase in PD-L1 expression, which was reversible upon knockdown IGFBP3. Mechanistically, IGFBP3 activated the JAK2/STAT3 signaling pathway, leading to an increase in PD-L1 expression. Additionally, co-culture experiments results showed IGFBP3 overexpression induced upregulation of PD-L1 expression promoted apoptosis in Jurkat cells, and this effect was blocked by IGFBP3 antibody and PDL-1 inhibitors. Importantly, in vivo experiments targeting IGFBP3 suppressed tumor growth and significantly prolonged the survival of mice.

**Conclusions:**

This research demonstrated IGFBP3 is a novel regulator for PD-L1 expression in GBM, and identified a new mechanism by which IGFBP3 regulates immune evasion through PD-L1, suggesting that IGFBP3 may be a potential novel target for GBM therapy.

**Supplementary Information:**

The online version contains supplementary material available at 10.1186/s12935-024-03234-3.

## Background

Glioma is a commonly occurring tumor in the central nervous system and is categorized into I–IV grades based on The World Health Organization (WHO) classification. Grade IV, known as GBM, is the most aggressive of the gliomas associated with poor prognosis and highly immunosuppressive microenvironment [1, 2]. The primary approach for treating glioma typically involves surgical removal, followed by postoperative radiation therapy and temozolomide-based chemotherapy [3]. However, the current effectiveness of this treatment remains unsatisfactory. The internal microenvironment of gliomas presents a distinct and complex state that lacks accurate and sensitive biomarkers for effective targeted therapy [4–6]. Thus, studying the molecular mechanism of GBM occurrence is crucial for improving therapy of patients.

IGFBP3 is a commonly IGF-specific binding protein used to stabilize IGF in blood, and it is involved in cell proliferation and apoptosis processes by IGF-dependent or other ligand-dependent mechanisms [7, 8]. IGFBP3, a protein that exhibits multiple regulatory functions, has elevated expression in multiple cancer types and assumes a decisive role in the malignant advancement of tumors [9–12]. The tumor cells expression and blood serum levels of IGFBP3 are related to the survival of GBM patients [13]. Nevertheless, the exact mechanism by which IGFBP3 is engaged in all this is still puzzling. Reportedly, there was a significant correlation between CUL4B and IGFBP3 in mesothelioma [14]. Besides, CUL4B deficiency affects immune cell function and infiltration [15, 16]. Based on the above research, we assumed that IGFBP3 possibly has an immunomodulatory role in GBM.

In recent years, immunotherapy has gained substantial attention for promoting anti-tumor immune reactions, which is an appealing approach to treat GBM [17]. The tumor microenvironment is complex and dynamic, and the excessive expression of immune checkpoints stands out as a primary property of tumor cells in evading the immune system [18, 19]. PD-1/PD-L1 is a considerably researched immune checkpoint pathway that plays a crucial role in inhibiting immune responses within the tumor microenvironment [20]. PD-L1 is known to inhibit the human immune response, making it a key player in regulating immune system activity. Additionally, it serves as a significant biomarker for predicting the efficacy of immunotherapy in various cancer checkpoint pathways [21–23]. Although antibody-targeted therapy against PD-1/PD-L1 has shown positive outcomes in inducing anti-tumor immune responses and treating various cancers, its effectiveness in GBM patients remains uncertain for now. Hence, understanding the specific mechanism of PD-L1 regulation in GBM is necessary in order to discover novel therapeutic approaches to improve the efficacy of immune checkpoint inhibitors.

In the present study, we demonstrated that IGFBP3 promotes the expression of PD-L1 by activating JAK2/STAT3 phosphorylation. In-vitro co-culture system comprised of GBM and Jurkat cells showed IGFBP3 induced upregulation of PD-L1 expression in GBM cells promoted the apoptosis of Jurkat cells. Additionally, knockdown of IGFBP3 attenuates tumor growth and significantly increases the survival rate in a mouse orthotopic tumor model. Altogether, these results furnish a theoretical basis for overcoming the immune tolerance of IGFBP3 expression in GBM.

## Material and methods

### Cell lines and reagents

The GBM cells GL261, U87, U251, U118, T98G, U343, A172, LN229 and Jurkat cell were acquired from ATCC (Manassas, VA, USA). Cell culture was implemented as previously described [24]. Cytokine IGFBP3 was purchased from Pepro-Tech (Cat No. AF-100-08, Beijing, China). Human IGFBP-3 antibody purchased from Bio-Techne (Cat No. AF675-SP, R&D Systems, USA). JAK2/STAT3 inhibitor WP1066 (Cat No. S2796, Selleck China) and PD-L1 inhibitor BMS-1166 (Cat No. HY-102011, MCE, China) were dissolved in DMSO (Cat No. D8371, Solarbio, China) and stored at − 20 °C according to the manufacturer’s instructions.

### Cell viability assay

Cells viability was assessed by Cell Counting Kit-8 (CCK-8) assay (Elabscience, E-CK-A362). In brief, CCK-8 reagent was added to each well and placed in a CO_2_ incubator for 4 h. The absorbance was measured at 450 nm using a microplate device (DMI3000, Leica, Germany).

### Apoptosis assay

Briefly, after co-culture with tumor cells, Jurkat cells were collected and stained with annexin V-FITC and PI (Elabscience, E-CK-A211). After incubation for 20 min at room temperature, apoptosis rate was measured using FCM (Beckman, USA).

### siRNA transfection

The siRNA targeting IGFBP3 and STAT3 were acquired from Genepharma (Shanghai, China). A mixture of siRNA and Lipofectamine 2000 (Cat No. 11668–019, Invitrogen, USA) were introduced to cells, after 48 h of infection, proteins were extracted to detect the transfection effect. si-IGFBP3: 5′-CAGAGCACAGAUACCCAGAACUU CU-3′, si-STAT3: 5′-CCCGGAAATTTAACATTCT-3′, si-NC: 5′-UAACGACGCG ACGACGUAA-3′.

### RNA extraction and qRT-PCR

QRT-PCR analysis as previously described [24]. The primer sequences are listed in Additional file 1: Table S1.

### Immunoblotting assay

Immunoblotting analysis as previously described [24]. All antibodies used are listed in Additional file 2: Table S2.

### Hematoxylin–Eosin (HE) and staining Immunohistochemistry (IHC)

For HE and IHC staining, after a 24h soak in paraformaldehyde, tumor brain tissue embedded in paraffin, and a set of 5 μm-thick slices were sliced. Dewaxing using xylene is followed by rehydration with a series of ethanol grades. Slices were soaked in an EDTA antigen-repair solution (pH 9.0) at high temperature for 30 min. After allowing the slices to be returned to ambient temperature, they were submerged in a 0.3% hydrogen peroxide solution for 15 min to suppress endogenous peroxidase activity. To block non-specific antibody binding sites, the slices were incubated at 37℃ for 15 min in goat serum with the same source of secondary antibodies. Subsequently, slices were treated with anti-IGFBP3 (1:50), anti-PD-L1 (1:500), and anti-CD31 (1:200), and then stained with diaminobenzidine (DAB, Cat No. GK600505, Gene Tech, China) for color development after incubation with biotin-labeled secondary antibody at 37 °C for 30 min. The slices were counterstained by hematoxylin and an Olympus IX81 microscope was used for observation.

### Immunofluorescence

After 48 h of cell culture on glass slices in 12-well plates, the culture medium was discarded, and the cells were fixed in 4% paraformaldehyde for 15 min. Flushed 3 times with TBST and then closed with a closure solution (containing 1% BSA, 4% goat serum, and 1% Triton X-100) for 1 h. Cells were incubated with IGFBP3 (1:50) and PD-L1 (1:500) overnight at 4 °C, and then secondary antibodies fluorescently labeled with FITC or Alexa were incubated with cells for 1 h. DAPI (Cat No. C1002, Beyotime Biotechnology, China) was used to stain the cell nucleus. Observed and photographed using a ZEISS LSM 510 META confocal microscope (Carl Zeiss, Jena, Germany).

### Lentivirus infection

Packaged IGFBP3 overexpression and sh-IGFBP3 lentiviral supernatants purchased from GeneCopoeia (Guangzhou, China). Purified lentiviral particles were used for transduction of U251 and U118 cells and purified sh-IGFBP3 lentiviral particles were used to infect LN229, T98G and GL261 cells. Lentiviral supernatant was added to cells at a density of 30% to 40%, and 72 h later, 2 μg/mL of puromycin (Cat No. 540222, Sigma-Aldrich) was used to screen infected cells. The following shRNA were used: sh-con: 5′-TTCTCCGAACGTGTCACGTTT-3′; sh-IGFBP3: 5′-CAGAGCACAGATACCCG AACTTCT-3′.

### IL-2 and IFN-γ ELISA

Human IL-2 (Cat No. EK102-48, MultiSciences, China) and IFN-γ (Cat No. EK180-48, MultiSciences, China) ELISA kits were used to detect the levels of IL-2 and IFN-γ in the cell culture supernatant according to the manufacturer’s instructions.

### Mice orthotopic tumor model

4-week-old female C57BL/6 J mice were acquired from Jinan Pengyue (Jinan, China). The mice were randomly divided into two groups (n = 20 per group), one group was used to observe tumor growth and the other group was used to monitor the survival time of the mice. 1 × 10^6^ of GBM cells (GL261-shcon, GL261-shIGFBP3) were injected into the caudate nucleus of mouse using a stereotactic device. Mice were given normal care during tumorigenesis. After 3 weeks, the mice were put to death and brain tissue was extracted in order to measure the tumor size. In addition, when animals were unable to feed ordinarily or lost over 20% of their body weight, they were euthanized and their survival times were recorded. The Ethics Committee of Binzhou Medical University approved all the procedures.

### RNA-sequencing and data collection

U251-vector and U251-IGFBP3 cells total RNA was extracted using the TRIzol reagent (Cat No. 15596018, Invitrogen, CA, USA). Then the libraries were constructed using VAHTS Universal V6 RNA-seq Library Prep Kit according to the manufacturer’s instructions. The transcriptome sequencing and analysis were conducted by OE Biotech Co., Ltd. (Shanghai, China). The libraries were sequenced using the llumina Novaseq 6000 sequencing platform and 150 bp double-ended reads were generated. About 49.5 M raw reads for each sample were generated. Raw reads of fastq format were firstly processed using fastq and the low-quality reads were removed to obtain the clean reads. Then about 48.5 M clean reads for each sample were retained for subsequent analyses. The clean reads were mapped to the human reference genome using HISAT2. Fragments Per Kilobase of exon model per Million mapped fragments (FPKM) of each gene was calculated and the read counts of each gene were obtained by HTSeq-count. Differential expression analysis was performed using the DESeq2. Q value < 0.05 and foldchange > 1.5 was set as the threshold for significantly differential expression gene (DEGs). The RNA-sequencing data are available at NCBI: PRJNA1003419.

Gene expression data and clinical data for glioma patients were obtained from the TCGA (http://cancergenome.nih.gov) and CGGA (http://www.cgga.org.cn) databases. The TCGA database collected 418 LGG and 152 GBM samples. In the CGGA database, 172 LGG and 237 GBM samples were available.

### Statistical analyses

The results of the experimental data are displayed as mean ± standard deviation. SPSS 23.0 was utilized for the purpose of statistical analysis, while GraphPad Prism 8 was employed to generate graphs. For the correlation analysis, the Spearman rank correlation test was utilized, and for the survival analysis, the Kaplan–Meier test and the log-rank test were utilized. A P value < 0.05 was deemed statistically significant.

## Results

### IGFBP3 positively correlates with PD-L1 expression in glioma patients

**Fig. 1:**
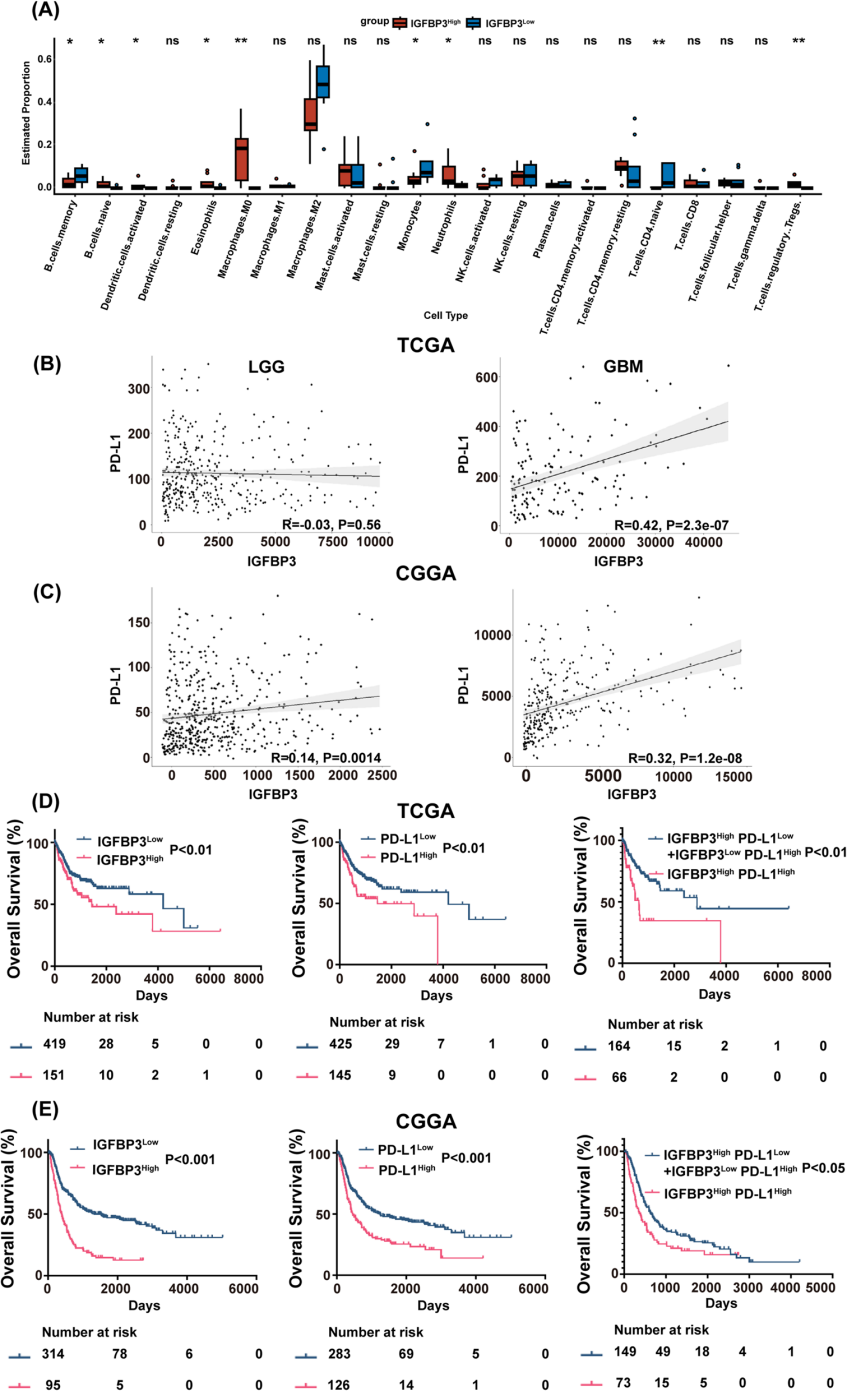
IGFBP3 positively correlates with PD-L1 expression in glioma patients. **A** The fraction of immune cells in the IGFBP3^Low^ and IGFBP3^High^ groups was assessed using CIBERSORT. **B**, **C** Correlation analysis of IGFBP3 and PD-L1 in TCGA (**B**) and CGGA (**C**) databases, left: LGG, right: GBM, and statistical significance was assessed using the spearman’s rank test. **D**, **E** Kaplan—Meier plots of IGFBP3 and PD-L1 gene expression in glioma patients from TCGA (**D**) and CGGA (**E**) databases, and statistical significance was assessed using the log-rank test

To determine whether IGFBP3 is related to the regulation of glioma immune microenvironment, we comprehensively analyzed immune cells infiltration in gliomas using the CIBERSORT algorithm. We divided the glioma samples into IGFBP3^Low^ and IGFBP3^High^ groups using the CGGA database (n = 325), and the status of 22 types of immune cells infiltration was compared. The results indicated that naive CD4 T cells, memory B cells and monocytes were obviously decreased in IGFBP3^High^ group, whereas regulatory T cells were significantly enriched (Fig. 1A). Therefore, we hypothesized that IGFBP3 may be involved in the immunosuppressive microenvironment of gliomas. Considering the crucial role of PD-L1 in immunosuppression. Next, to investigate the relationship between IGFBP3 and the immune checkpoint ligand PD-L1, we collected gene expression data of IGFBP3 and PD-L1 of patients diagnosed with LGG (n = 418) and GBM (n = 152) from the TCGA. Our results indicated that in GBM patients, IGFBP3 expression was positively correlated to PD-L1 expression (R = 0.42, P = 2.3e−07). However, in LGG patients, there was no clear correlation observed between IGFBP3 and PD-L1 expression levels (R = − 0.03, P = 0.56) (Fig. 1B). In the CGGA database, IGFBP3 and PD-L1 gene expression data of LGG patients (n = 172) and GBM patients (n = 237) were also collected. Analysis of the data showed that the expression levels of IGFBP3 and PD-L1 have a low association in LGG (R = 0.14, P = 0.0014) while they have high association in GBM patients (R = 0.32, P = 1.2e−08) (Fig. 1C). Following, our investigation aimed to determine if there existed a correlation between IGFBP3/PD-L1 gene expression and the overall survival (OS) of individuals with gliomas. Our results indicated that glioma patients with high expression of IGFBP3 or PD-L1 had a lower survival. Subsequently, we divided all patients with combined IGFBP3 and PD-L1 expression into two subgroups. The data from our study clearly confirmed that patients with high gene expression of both IGFBP3 and PD-L1 had markedly worse OS compared to those with elevated expression alone (Fig. 1D). These results were also consistently observed in CGGA dataset (Fig. 1E). Taken together, these results suggested that IGFBP3 expression was positively correlated with PD-L1 expression in GBM.

### IGFBP3 transcriptionally regulates PD-L1 expression in GBM cells

**Fig. 2:**
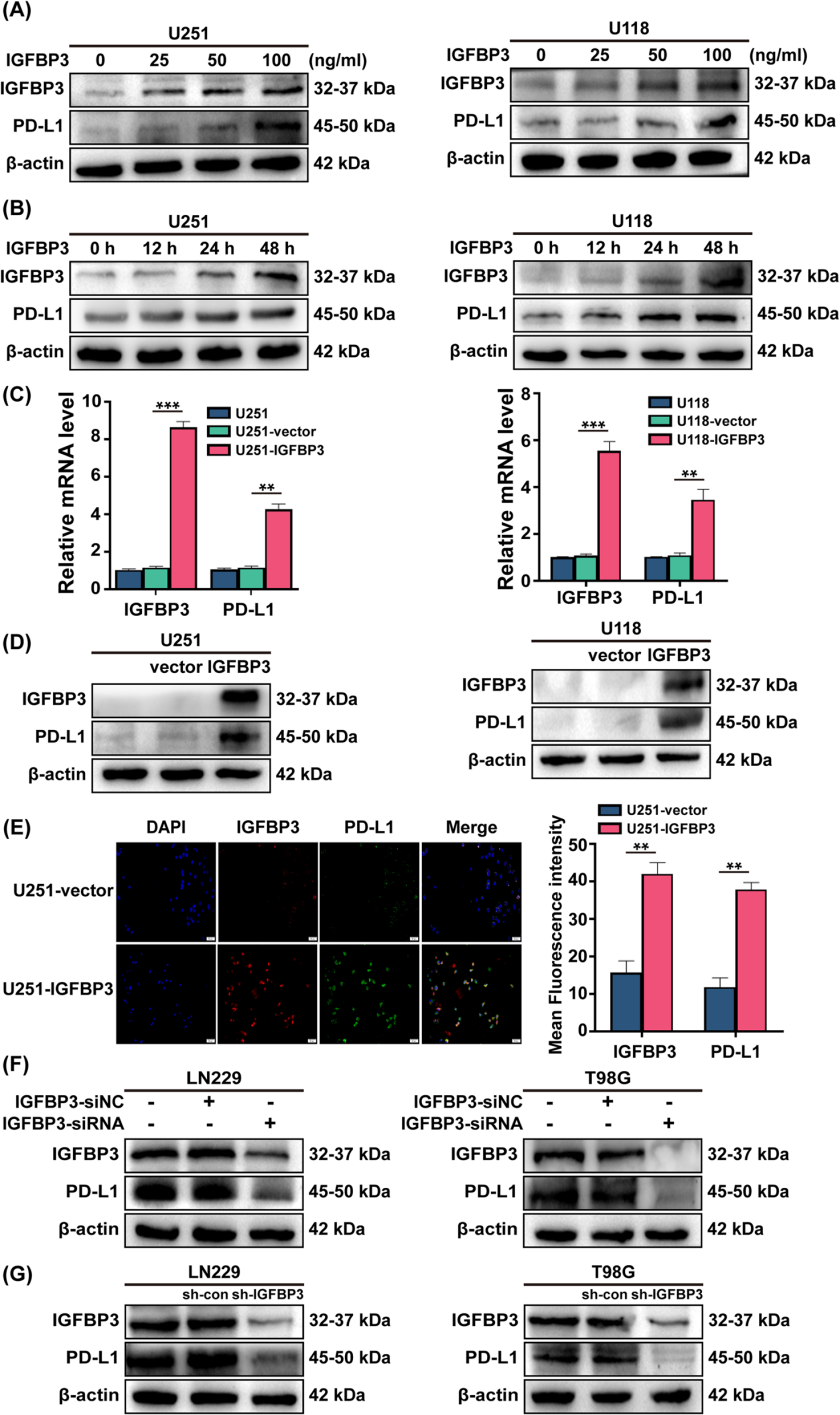
IGFBP3 transcriptionally regulates PD-L1 expression in GBM cells. **A** Immunoblotting analysis of IGFBP3 and PD-L1 expression in U251 and U118 cells treated with different concentrations of exogenous IGFBP3 (0, 25, 50, and 100 ng/mL) for 24 h. **B** Immunoblotting was performed to determine the expression of IGFBP3 and PD-L1 in U251 and U118 cells stimulated with 100 ng/mL of IGFBP3 for 0, 12, 24, and 48 h. **C** QRT-PCR analysis of IGFBP3 and PD-L1 mRNA expression levels in U251 and U118 cells infected with overexpressing IGFBP3 and empty vector lentivirus. **D** Immunoblotting assay of IGFBP3 and PD-L1 protein expression in U251 and U118 cells infected with overexpressing IGFBP3 and empty vector lentivirus. **E** Representative immunofluorescence images of DAPI (blue), IGFBP3 (red), PD-L1 (green) in U251-vector and U251-IGFBP3 cells. **F** Immunoblotting was performed to examine the expression of IGFBP3 and PD-L1 in LN229 and T98G cells transfected with si-RNA targeting IGFBP3 (si-IGFBP3) and negative control (si-NC). **G** Immunoblotting analysis of IGFBP3 and PD-L1 expression in LN229 and T98G cells infected with lentivirus of targeting IGFBP3 (sh-IGFBP3) and control (sh-con). Data are expressed as mean ± SD. **P < 0.01; ***P < 0.001, Student *t* test

**Fig. 3:**
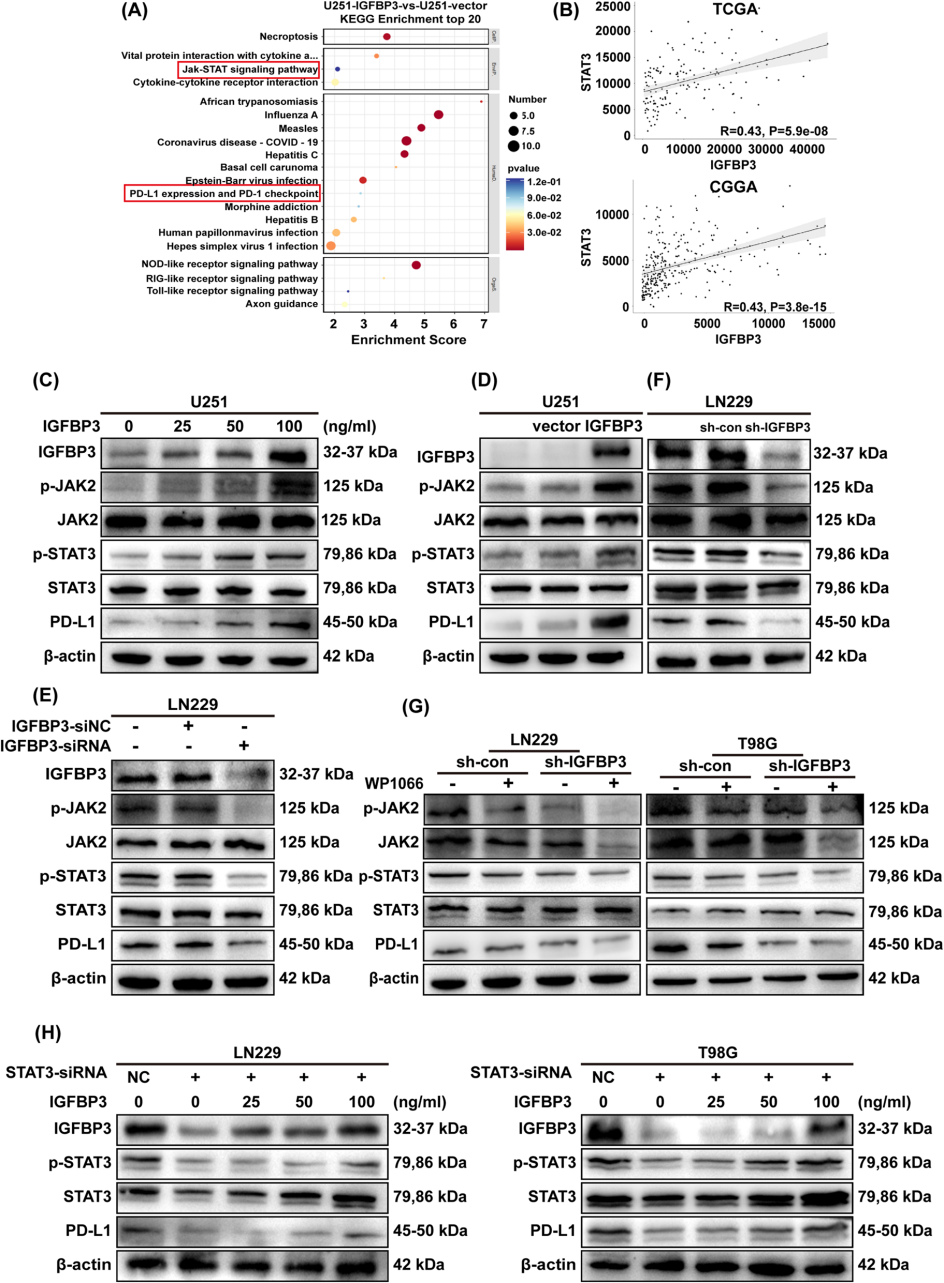
IGFBP3 increases PD-L1 expression by up-regulating the phosphorylation of STAT3 in GBM cells. **A** KEGG analysis of differential genes. **B** Correlation analysis of IGFBP3 and STAT3 in TCGA and CGGA databases, and statistical significance was assessed using the spearman’s rank test. **C** Immunoblotting analysis of IGFBP3, p-JAK2, p-STAT3, and PD-L1 expression in U251 cell treated with different concentrations of IGFBP3 for 24 h. **D** Immunoblotting analysis of IGFBP3, p-JAK2, p-STAT3, and PD-L1 expression in U251 cells infected with IGFBP3 overexpressing and empty vector lentivirus. **E** Immunoblotting was performed to examine the expression of IGFBP3, p-JAK2, p-STAT3, and PD-L1 in LN229 cell transfected with si-IGFBP3 and si-NC. **F** Immunoblotting analysis of IGFBP3, p-JAK2, p-STAT3, and PD-L1 expression in LN229 cell infected with sh-IGFBP3 and sh-con. **G** LN229 and T98G cells with IGFBP3 knockdown were treated with the JAK2/STAT3 inhibitor WP1066 for 24 h, cell lysate was used to analyze the expression of p-JAK2, p-STAT3 and PD-L1. **H** Immunoblotting was used to analyze the expression of IGFBP3, p-STAT3, and PD-L1 in STAT3-silenced LN229 and T98G cells stimulated with different concentrations of exogenous IGFBP3 for 24 h

To gain more insight into the association between IGFBP3 and PD-L1 in GBM, we added varying concentrations of exogenous IGFBP3 to U251 and U118 cells for 24 h, which lowly express endogenous IGFBP3 (Additional file 3: Fig. S1A). The results showed that the expression of IGFBP3 and PD-L1 increased dose-dependent manner in response to IGFBP3 stimulation (Fig. 2A). Following that, 100 ng/mL IGFBP3 was chosen and added to U251 and U118 cells at different time periods, and the findings displayed a time-dependent manner of increased in IGFBP3 and PD-L1 expression (Fig. 2B). On the basis of these results, it is hypothesized that IGFBP3 may play a role in modulating PD-L1 expression in GBM cells. To provide direct evidence that IGFBP3 could regulate PD-L1 expression, we infected U251 and U118 cells with IGFBP3 overexpressing lentiviral supernatants. The results of qRT-PCR and immunoblotting indicated the established stable overexpress IGFBP3 cell lines U251-IGFBP3 and U118-IGFBP3 (Additional file 3: Fig. S1B-C). We used qRT-PCR and immunoblotting to examine the expression of IGFBP3 and PD-L1 in IGFBP3 overexpressed GBM cells. Both qRT-PCR and immunoblotting data supported our hypothesis that PD-L1 expression is elevated in U251-IGFBP3 and U118-IGFBP3 cells (Fig. 2C, D). Moreover, immunofluorescence showed enhanced fluorescence intensity of PD-L1 in U251-IGFBP3 cell (Fig. 3E). Consistent data were observed in U118-IGFBP3 cell (Additional file 3: Fig. S1D). Following, we attenuated IGFBP3 expression in LN229 and T98G cells using si-RNA and sh-RNA targeting IGFBP3, which highly express endogenous IGFBP3 (Additional file 3: Fig. S1A). Immunoblotting was used to detect knockdown efficiency of IGFBP3, and data demonstrated the LN229-shIGFBP3 and T98G-shIGFBP3 cell lines had been stable establishment (Additional file 3: Fig S1E-F). The results revealed that si-RNA targeting IGFBP3 decreased PD-L1 expression (Fig. 2F). Consistent data were observed in LN229-shIGFBP3 and T98G-shIGFBP3 cells (Fig. 2G). To put it briefly, IGFBP3 is a key factor regulating PD-L1 expression in GBM cells.

### IGFBP3 increases PD-L1 expression by up-regulating the phosphorylation of JAK2/STAT3 in GBM cells

To understand the potential mechanism by which IGFBP3 regulates PD-L1 expression in GBM, U251-vector and U251-IGFBP3 cells were subjected to RNA-seq to find out the differential gene, and these differential genes were enriched by KEGG pathway. The JAK-STAT signaling pathway was at the top of the enriched pathways among them (Fig. 3A). Notably, we also observed significant enrichment of the PD-1/PD-L1 signaling pathway (Fig. 3A). Moreover, using the TCGA and CGGA databases, it was discovered that expression of STAT3 and IGFBP3 was positively associated in GBM patients (Fig. 3B). To further verify that the JAK2/STAT3 is involved in IGFBP3 regulating PD-L1 expression, exogenous IGFBP3 was used to stimulate U251 cell. The immunoblotting revealed that IGFBP3 increased the phosphorylation level of JAK2/STAT3 in a dose-dependent and time-dependent manner (Fig. 3C and Additional file 4: Fig. S2A). The consistent findings were also obtained in U118 cells (Additional file 4: Fig. S2B-C). Next, to examine whether JAK2/STAT3 was involved in PD-L1 expression induced by IGFBP3 overexpression, U251-IGFBP3 and U118-IGFBP3 cells were used. We showed overexpression IGFBP3 also promoted the phosphorylation of JAK2/STAT3 (Fig. 3D and Additional file 4: Fig. S2D). To provide insight on the significant involvement of JAK2/STAT3 in the IGFBP3/PD-L1 axis, we interfered with IGFBP3 expression in LN229 and T98G cells using IGFBP3 siRNA. Immunoblotting confirmed that IGFBP3 siRNA markedly decreased IGFBP3 expression, and inhibited p-JAK2, p-STAT3 and PD-L1 expression (Fig. 3E and Additional file 4: Fig. S2E). Besides, in LN229-shIGFBP3 and T98G-shIGFBP3 cells the expression of p-JAK2, p-STAT3 and PD-L1 were also decreased (Fig. 3F and Additional file 4: Fig. S2F). More importantly, when the JAK2/STAT3 inhibitor WP1066 was added to IGFBP3 knockdown cells, PD-L1 expression was further reduced (Fig. 3G). Moreover, when STAT3 siRNA was transfected into sh-IGFBP3 cells, PD-L1 expression was extremely inhibited (Additional file 4: Fig. S2G). Next, to investigate the relationship between PD-L1 expression in response to STAT3 silencing and the dose of exogenous IGFBP3, different concentrations of exogenous IGFBP3 were added to STAT3-silenced and control cells. Immunoblotting results showed that silencing STAT3 inhibited the expression of PD-L1 in LN229 and T98G cells, but this effect was gradually abolished with the increase of exogenous IGFBP3 concentration (Fig. 3H). Overall, our experimental results suggested that JAK2/STAT3 participates in the IGFBP3 modulation of PD-L1 expression in GBM cells.

### IGFBP3 induces immunosuppression in in-vitro co-culture system

**Fig. 4:**
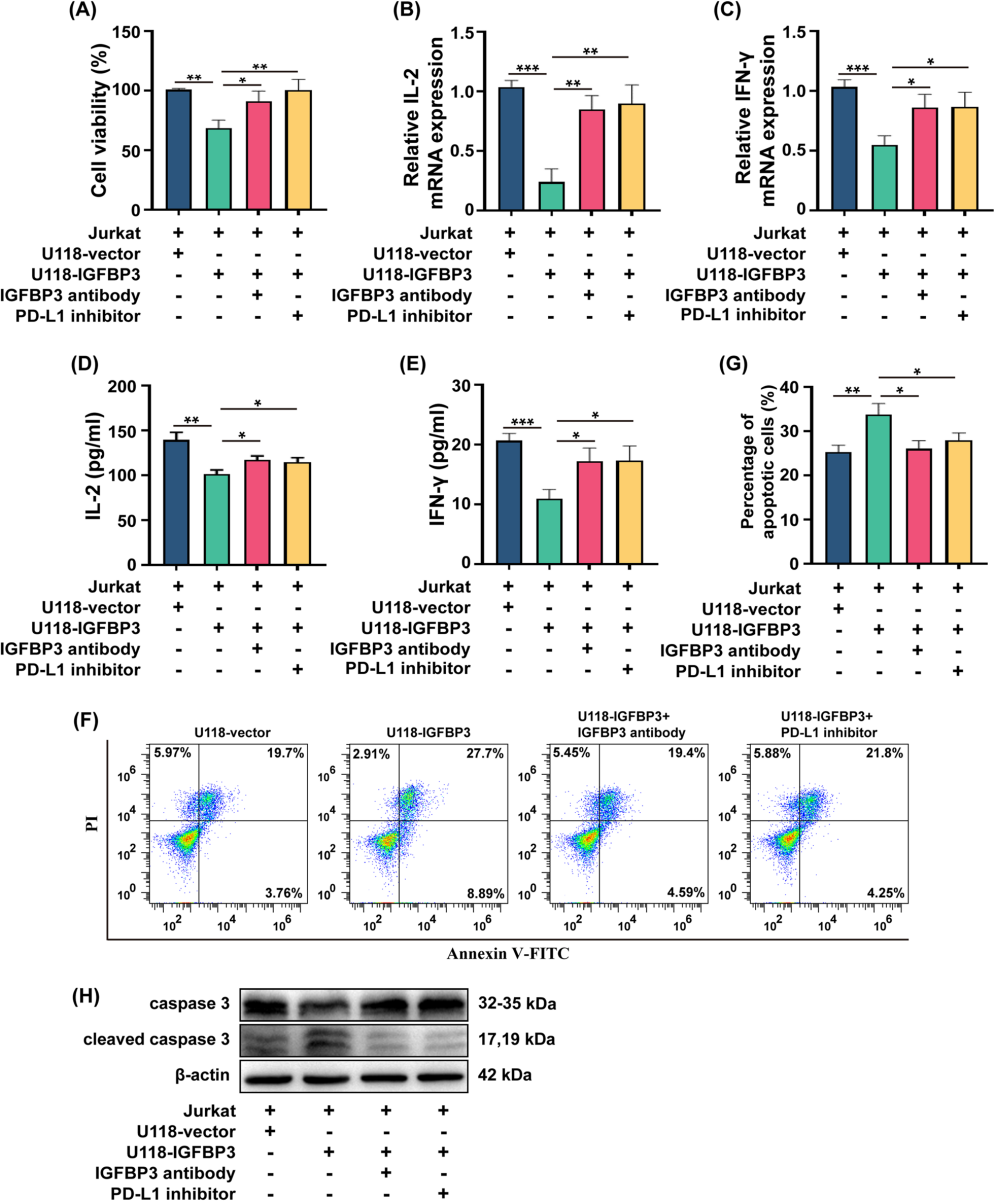
IGFBP3 induces immunosuppression in in-vitro co-culture system. **A** CCK-8 assay was used to analysis the viability of Jurkat cells after co-cultured with IGFBP3 overexpressing cells. **B**, **C** QRT-PCR was performed to examine the mRNA expression of IL2 (**B**) or IFN-γ (**C**) in Jurkat cells after co-cultured with IGFBP3 overexpressing cells. **D**, **E** ELISA was used to measure the levels of IL-2 (**D**) and IFN-γ (**E**) in the supernatants of Jurkat cells after co-cultured with IGFBP3 overexpressing cells. **F** Annexin V-PE apoptosis assay was used to determine apoptosis of Jurkat cells after co-cultured with IGFBP3 overexpressing cells. **G** Statistical chart of apoptotic cells. **H** Immunoblotting was performed to examine the caspase3 expression in Jurkat cells after co-cultured with IGFBP3 overexpressing cells. Data are expressed as mean ± SD. *P < 0.05; **P < 0.01; ***P < 0.001, Student *t* test

To investigate the effect of IGFBP3 regulated PD-L1 expression on T cells viability, we established a co-culture system of tumor cells with Jurkat cells that had been activated with phorbol 12 myristate 13 acetate (PMA, 20 ng/ml) and ionomycin (500 ng/ml) for 12 h at a ratio of 4:1 (T cells: tumor cells) for 24 h in vitro. After co-culture, the proliferation of Jurkat cells was detected by CCK-8 assay. The results showed that the viability of Jurkat cells was impaired by co-culture with overexpressing IGFBP3 tumor cells, which was blocked by IGFBP3 antibody and PD-L1 inhibitor (Fig. 4A). Activated T cells secrete Interleukin 2 (IL-2), which promotes the proliferation and differentiation of T cells [25, 26]. Interferon-γ (IFN-γ) is secreted by activated T cells and exerts anti-tumor effects [27]. In order to detect the effect of IGFBP3 on Jurkat cells activation, we measured the mRNA expression of IL-2 and IFN-γ in Jurkat cells after co-culture with IGFBP3 overexpressing cells. The results indicated that the levels of IL-2 and IFN-γ mRNA expression in Jurkat cells were suppressed following co-culture with cells overexpressing IGFBP3, but rescued by IGFBP3 antibody and PD-L1 inhibitor (Fig. 4B, C). To further investigate the changes in IL-2 and IFN-γ proteins secreted by Jurkat cells after co-culture with cells overexpressing IGFBP3, ELISA was used to detect the levels of IL-2 and IFN-γ in the supernatants. The results of ELISA were consistent with those obtained from qRT-PCR (Fig. 4D, E). In addition, the apoptosis of Jurkat cells after co-culture with IGFBP3 overexpressing cells was detected by Annexin V-PI assay. The results showed that overexpression of IGFBP3 induced apoptosis in Jurkat cells after co-culturing with Jurkat cells, and it was blocked by IGFBP3 antibody and PD-L1 inhibitor (Fig. 4F, G). In addition, we also detected the apoptosis-related protein caspase3 using immunoblotting. The results showed that the expression of caspase3 was reduced and cleaved caspase3 was increased in Jurkat cells after co-culture with cells overexpressing IGFBP3, and IGFBP3 antibody and PD-L1 inhibitor could block this process (Fig. 4H). Collectively, these results indicate that IGFBP3 induced upregulation of PD-L1 could lead to immunosuppression of T cells.

### IGFBP3 targeting therapy efficiently suppresses glioblastoma invasion in vitro and tumor growth in vivo via reducing PD-L1 expression

**Fig. 5:**
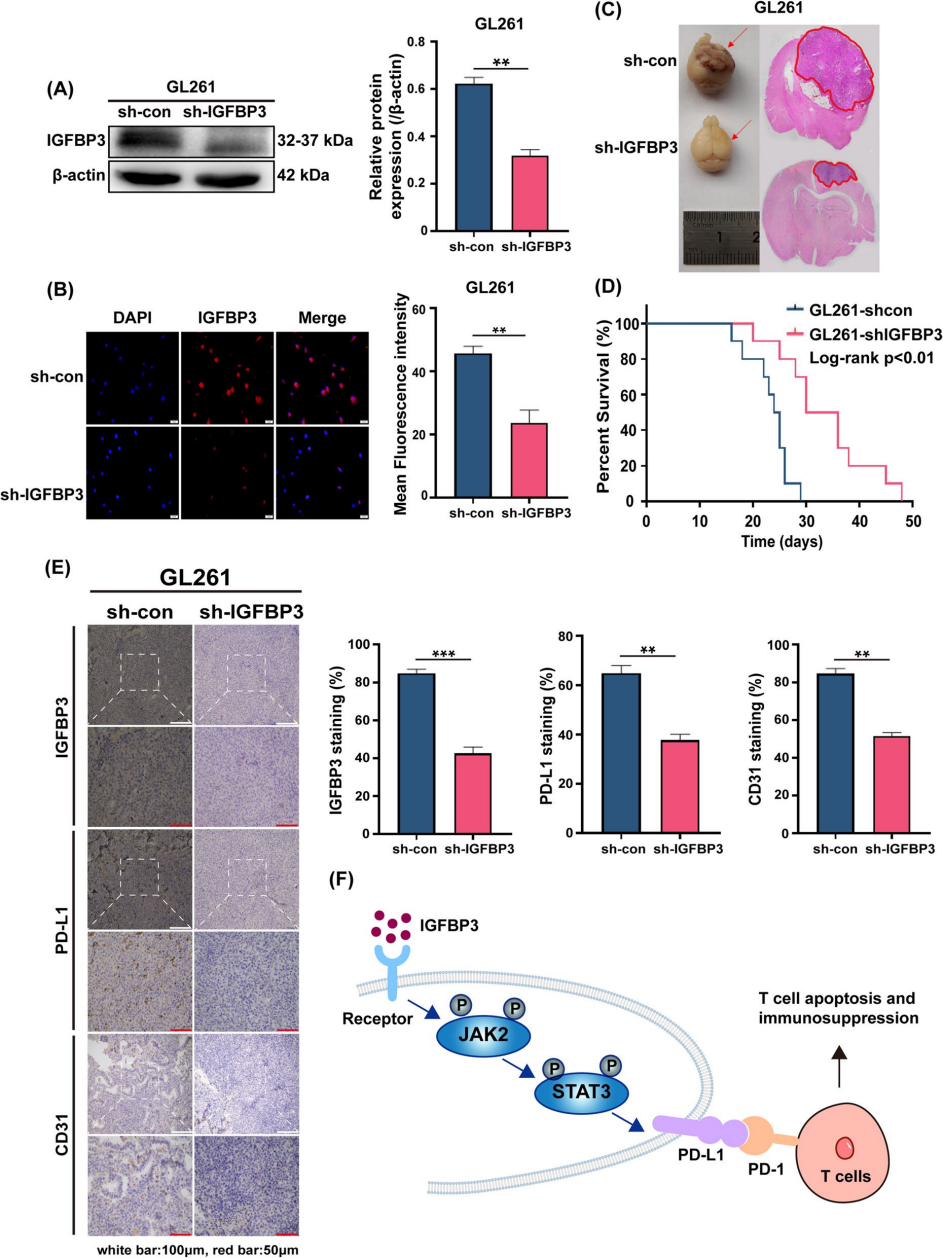
Targeting IGFBP3 therapy efficiently suppresses glioblastoma invasion in vitro and tumor growth in vivo via reducing PD-L1 expression. **A** Immunoblotting analysis of IGFBP3 expression in GL261 cell infected with sh-IGFBP3 and sh-con. **B** Immunofluorescence was used to examine IGFBP3 expression in GL261 cell infected with sh-IGFBP3 and sh-con. **C** Left: Representative image of brain orthotopic tumor. Right: HE stained representative image of brain tumor section. **D** Kaplan–Meier plots for GL261-shcon and GL261-shIGFBP3 groups, and statistical significance was assessed using the log-rank test. **E** Immunohistochemical representative images of IGFBP3, PD-L1 and CD31 in the GL261-shcon and GL261-shIGFBP3 groups. **F** IGFBP3 up-regulates the expression of PD-L1 by activating JAK2/STAT3 signaling pathway. PD-L1 binds to T cell–derived PD-1 to promote T cell apoptosis and immunosuppression. Data are expressed as mean ± SD. **P < 0.01; ***P < 0.001, Student *t* test

Since upregulation of IGFBP3 was associated with poor patient prognosis, we further explored its biological role in tumors. First, in vitro experiments on two GBM cell lines with IGFBP3 knockdown demonstrated that the suppression of IGFBP3 inhibited the proliferation and invasion of GBM cells (Additional file 5: Fig. S3). Next, we investigated the tumor-promoting role of IGFBP3 in vivo. Stable knockdown of IGFBP3 cell line GL261-shIGFBP3 was established using shRNA of IGFBP3 to infect GL261 cell. Immunoblotting and immunofluorescence confirmed that IGFBP3 shRNA greatly reduced IGFBP3 expression in GL261-shIGFBP3 cell (Fig. 5A, B). We established C57BL/6J mice brain orthotopic tumor model, observed that knockdown of IGFBP3 attenuated the growth of tumors at 21 days after tumor cell injection (Fig. 5C), and significantly improved the survival rate of mice (Fig. 5D). Moreover, immunohistochemical results indicated that knockdown of IGFBP3 inhibited PD-L1 and CD31 expression in tumors (Fig. 5E). In summary, our findings show that knockdown of IGFBP3 attenuates in brain orthotopic tumor formation, and prolongs the survival of mice.

## Discussion

The GBM microenvironment is a complex network of immunosuppressive neuroinflammation [2], which is characterized by an enrichment of suppressor immune cells but the absence of T cells infiltration [28]. The use of antibodies against PD-1/PD-L1 in targeted therapy has demonstrated significant potential in various types of cancer, such as breast cancer [29, 30], colorectal cancer [31], lung cancer [32] and osteosarcoma [33]. However, a stage III trial comparing nivolumab (anti-PD-1 monoclonal antibody) and bevacizumab (anti-VEGF monoclonal antibody) in 369 patients with recurrent GBM did not prove the benefit of nivolumab [34]. At the same time, one study offered proof that anti-PD-1 rescue treatment did not result in a treatment advantage in individuals suffering from recurrent high-grade gliomas [35]. Therefore, it is extremely significant to elucidate how PD-L1 expression is regulated in GBM. There are various factors that impact PD-L1 expression, such as genetic aberrations, mRNA regulation, oncogenic, and inflammatory signaling [36]. In gliomas, orphan nuclear receptors TLX [37], and ALKBH5 [38] have been described to increase PD-L1 expression. Meanwhile, the investigation of new mechanisms for modulating the PD-L1 checkpoint still remains an appealing area of research that may have significant effects on better PD-1/PD-L1 therapy. Our research presents fresh evidence indicating a positive association between IGFBP3 and PD-L1 expression. Furthermore, we have discovered that IGFBP3 plays a regulatory role in PD-L1 expression through the JAK2/STAT3 signaling pathway. In brief, our finding uncovered a novel mechanism of PD-L1 regulation in GBM. The results of the present study showed that IGFBP3 activates phosphorylation of JAK2/STAT3 to induce PD-L1 expression, thereby promoting GBM immune escape.

According to various studies, the expression of IGFBP3 has been found to be associated with multiple types of cancer and exert different functions, which depend on the cellular environment and tumor type. Early studies have indicated that IGFBP3 suppresses the growth of lung cancer [39], liver cancer [40], and prostate cancer [41] cells. On the contrary, IGFBP3 is abundantly expressed in nasopharyngeal carcinoma and is associated with poor prospects and tumor metastasis [10]. High expression of IGFBP3 can facilitate the growth of endophytic squamous cell carcinoma cells of the tongue [42] and stimulates human osteosarcoma cell migration [11]. In addition, the expression of IGFBP3 was also increased in gliomas, and associated with IDH1/2 mutations status, histology and tumor grade, it was suggested that IGFBP3 may be a prospective new target for the therapy of glioma [43]. Recently, a study reported that IGFBP3 may play a role in the immune microenvironment in hypertrophic cardiomyopathy [44], but its role in glioma immune microenvironment is still unclear. Our studies showed that naive CD4 T cell infiltration was reduced in the tumor microenvironment of glioma patients with high IGFBP3 expression. Since PD-L1 on the surface of tumor cells binds to PD-1 on T cells, it inhibits T cells proliferation and cytokine production [45, 46]. Based on previous studies and our preliminary results, therefore, we hypothesized that IGFBP3 performs an important role in regulating PD-L1 expression in gliomas. Encouragingly, on the basis of TCGA and CGGA database analysis, our results showed a positive association between IGFBP3 and PD-L1 expression in GBM, but no clear correlation in LGG. Furthermore, patients with high expression of both IGFBP3 and PD-L1 had lower survival rates compared to those with high expression of either IGFBP3 or PD-L1 alone. These results suggested that positive correlation of IGFBP3 with PD-L1 was associated with high grade glioma progression and poor prognosis. Our study further demonstrated that the overexpression of IGFBP3 induces an upregulation of PD-L1 expression in GBM cells, and this effect can be reversed by the knockdown of IGFBP3. Similarly, targeting IGFBP3 suppresses glioblastoma invasion in vitro and tumor growth in vivo by reducing PD-L1 expression. Importantly, the results of our in vitro co-culture system demonstrated that IGFBP3 up-regulates PD-L1 to inhibit the activation and promote the apoptosis of Jurkat cells, leading to immunosuppression, which is similar to the previously reported results that TLX [37], ALKBH5 [38] and β-catenin [47] promote immunosuppression and immune escape by facilitating PD-L1 expression in gliomas. Collectively, these results suggested that IGFBP3 induces upregulation of PD-L1 expression to promote apoptosis in Jurkat cells, leading to immune evasion of GBM.

Lately, some evidences have suggested that various transcription factors, such as JAK2/STAT3 [48, 49], NF-kB [50], PI3K/AKT/mTOR [51] and MEK1/2/ERK1/2 [52] facilitate the transcription of PD-L1 in cancer. Among them, JAK2/STAT3 is an essential oncogenic transcription factor and is the basis of multiple receptors signaling, which modulating target genes expression through DNA binding, nuclear translocation and phosphorylation activation [53]. Reportedly, FGFR2 contributes to activating the JAK2/STAT3 pathway in colorectal cancer, resulting in the promotion of PD-L1 expression [48]. However, it is unknown whether and how JAK2/STAT3 is engaged in the IGFBP3-PD-L1 regulatory axis. Therefore, we searched for the signaling pathways by which IGFBP3 regulates PD-L1 expression in GBM. We obtained the differentially expressed genes for the overexpressed IGFBP3 by RNA-seq and subjected the differentially expressed genes to KEGG pathway enrichment, which showed significant upregulation of JAK-STAT signaling pathway. Furthermore, in U251-IGFBP3 and U118-IGFBP3 cells, IGFBP3 caused increased p-JAK2 and p-STAT3, accompanied by increased PD-L1 expression, whereas this phenomenon was reversed when IGFBP3 was knocked down in LN229 and T98G cells. Additionally, our findings also showed that the use of the JAK2/STAT3 inhibitor WP1066 and the STAT3 siRNA further reduced PD-L1 expression, indicating the crucial role of the JAK2/STAT3 signaling pathway in the IGFBP3-PD-L1 axis. In summary, our results confirm that the JAK2/STAT3 signaling pathway is stimulated by IGFBP3, which upregulates PD-L1 expression.

## Conclusion

In conclusion, this study provides a new regulatory mechanism for PD-L1 expression in GBM. The expression of PD-L1 is regulated by IGFBP3 through the activation of JAK2/STAT3 phosphorylation. Additionally, we have demonstrated that IGFBP3 induces the expression of PD-L1 to promote immune evasion in GBM (Fig. 5F). Importantly, “IGFBP3 Targeting Therapy” efficiently suppresses GBM invasion in vitro and tumor growth in vivo via reducing PD-L1 expression, suggesting that IGFBP3 may be a potential novel target for GBM therapy.

### Supplementary Information

**Additional file 1: Table S1**. qRT-PCR primers.

**Additional file 2: Table S2**. Information about antibodies.

**Additional file 3: Fig. S1**. Immunoblotting was used to select cell lines and verify the effect of IGFBP3 overexpression or silencing.

**Additional file 4: Fig. S2**. Immunoblotting was used to confirm that IGFBP3 regulates PD-L1 expression through the JAK2/STAT3 signaling pathway.

**Additional file 5: Fig. S3**. Knockdown of IGFBP3 inhibits proliferation and invasion of LN229 and T98G cells.

## Data Availability

The datasets presented in this study can be found in online repositories. The names of the repository/repositories and accession number(s) can be found in the article / Supplementary Material.

## Abbreviations

CCK-8: Cell counting kit-8
CGGA: Chinese Glioma Genome Atlas
GBM: Glioblastoma
HE: Hematoxylin–eosin
IFN-γ: Interferon-γ
IGFBP3: Insulin-like growth factor binding protein 3
IL-2: Interleukin 2
JAK2: Janus Kinase 2
KEGG: Kyoto Encyclopedia of Genes and Genomes
LGG: Lower-grade glioma
OS: Overall survival
PD-1: Programmed cell death protein 1
PD-L1: Programmed death-ligand 1
qRT-PCR: Quantitative RT-PCR
RNA-seq: RNA sequencing
shRNA: Short hairpin RNA
siRNA: Small interfering RNA
STAT3: Signal transducer and activator of transcription 3
TCGA: The Cancer Genome Atlas
