# Supplementary material for: IGFBP3 induces PD-L1 expression to promote glioblastoma immune evasion
Source: Cancer Cell Int. 2024 Feb 7;24:60. doi: 10.1186/s12935-024-03234-3 (PMC10851611; doi:10.1186/s12935-024-03234-3)
Supplement: Supplementary file 1 — Additional file 1: Table S1. qRT-PCR primers. [file 12935_2024_3234_MOESM1_ESM.docx]

**Supplementary Table S1**

**qRT-PCR primers**

| **Primer** | **Forward (5’ to 3’)** | **Reverse (5’ to 3’)** |
| --- | --- | --- |
| GAPDH | GGAGCGAGATCCCTCCA AAAT | GGCTGTTGTCATACTTCT CATGG |
| IGFBP3 | AGAGCACAGATACCCAGAACT | GGT GATTCAGTGTGTCTTCCA |
| PD-L1 | TGGCATTTGCTGAACGCAT TT | TGCAGCCAGGTCTAATTGTTTT |
| IL-2 | GAATGGAATTAATAATTACAAGAATCCC | TGTTTCAGATC CCTTTAGTTCCAG |
| IFN-γ | GAGTGTGGAGACCATCAAGGAAG | TGCTTTGCGTTGGACATTCAAGTC |
