# Supplementary material for: IGFBP3 induces PD-L1 expression to promote glioblastoma immune evasion
Source: Cancer Cell Int. 2024 Feb 7;24:60. doi: 10.1186/s12935-024-03234-3 (PMC10851611; doi:10.1186/s12935-024-03234-3)
Supplement: Supplementary file 2 — Additional file 2: Table S2. Information about antibodies. [file 12935_2024_3234_MOESM2_ESM.docx]

Supplementary Table S2

Information about Antibodies

| **Antibody** | **Product Manufacturer** | **Dilutions** | **Cat No.** |  |
| --- | --- | --- | --- | --- |
| β-actin | Proteintech | 1:5000 | 66009-1-Ig |  |
| IGFBP3 | Proteintech | 1:800 | 10189-2-AP |  |
| PD-L1 | Proteintech | 1:2000 | 66248-1-Ig |  |
| STAT3 | Cell Signaling Technology | 1:1000 | #9139 |  |
| p-STAT3 | Cell Signaling Technology | 1:1000 | #9145 |  |
| JAK2 | Cell Signaling Technology | 1:1000 | #4406 |  |
| p-JAK2 | Cell Signaling Technology | 1:1000 | #3230 |  |
| Caspase3 | Proteintech | 1:2000 | 66470-2-Ig |  |
| Cleaved-Caspase3 | Proteintech | 1:1000 | 25128-1-AP |  |
| CD31 | Proteintech | 1:200 | 11265-1-AP |  |
| Anti-rabbit | Proteintech | 1:1000 | SA00001-2-AP |  |
| Anti-mouse | Proteintech | 1:1000 | SA00001-1-AP |  |
