# Supplementary material for: IGFBP3 induces PD-L1 expression to promote glioblastoma immune evasion
Source: Cancer Cell Int. 2024 Feb 7;24:60. doi: 10.1186/s12935-024-03234-3 (PMC10851611; doi:10.1186/s12935-024-03234-3)
Supplement: Supplementary file 3 — Additional file 3: Fig. S1. Immunoblotting was used to select cell lines and verify the effect of IGFBP3 overexpression or silencing. [file 12935_2024_3234_MOESM3_ESM.docx]

**Supplementary figure 1**

**
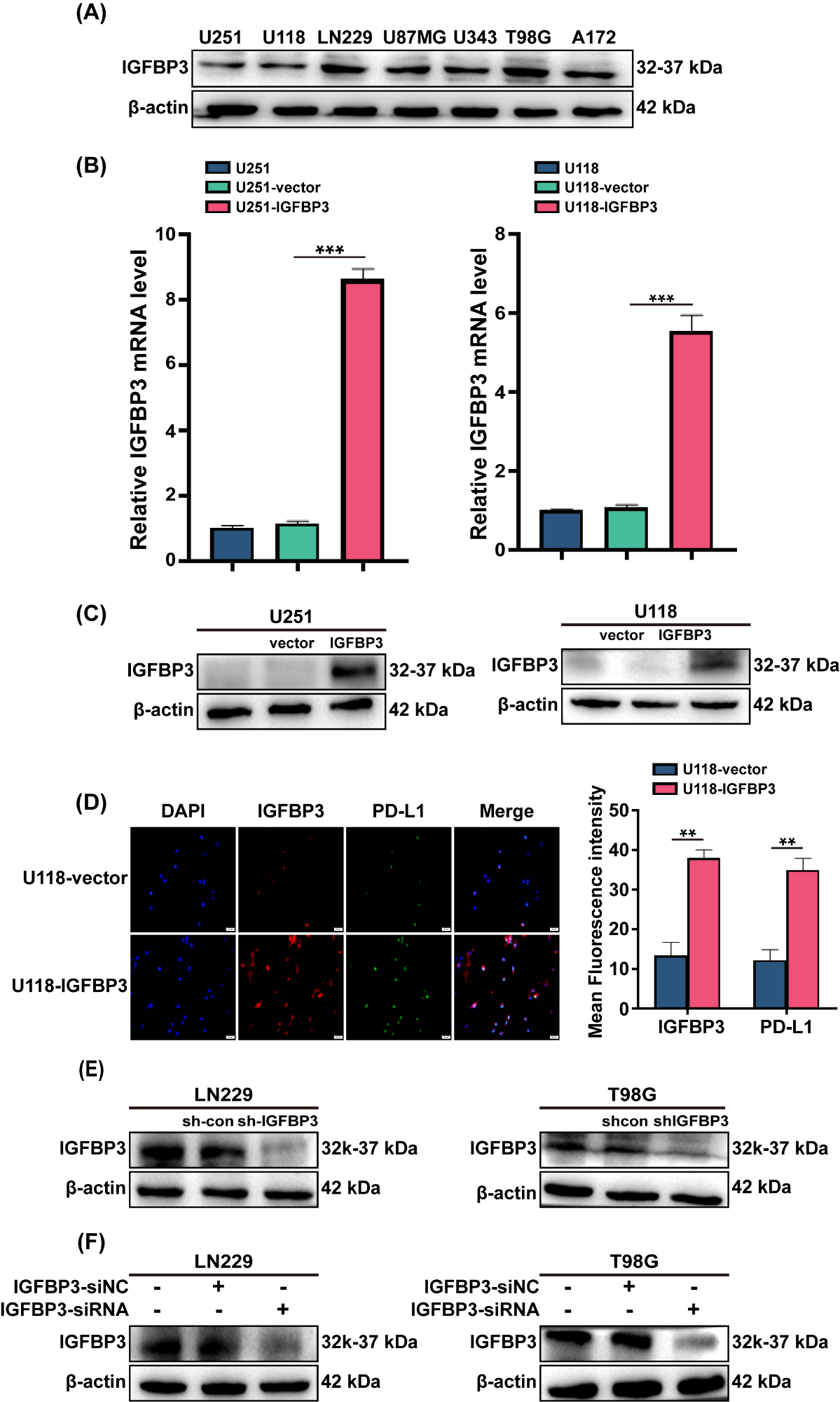
**

**Supplementary figure 1** Immunoblotting was used to select cell lines and verify the effect of IGFBP3 overexpression or silencing. **(A)** Immunoblotting was performed to examine IGFBP3 expression in different GBM cells. **(B)** qRT-PCR analysis of IGFBP3 mRNA expression levels in U251 and U118 cells infected with overexpressing IGFBP3 and empty vector lentivirus. **(C)** Detection of IGFBP3 expression in U251 and U118 cells infected with overexpressing IGFBP3 and empty vector lentivirus by immunoblotting. **(D)** Representative immunofluorescence images of DAPI (blue), IGFBP3 (red), PD-L1 (green) in U118-vector and U118-IGFBP3 cells, U118-vector: U118 cell infected with empty vector lentivirus, U118-IGFBP3: U118 cell infected with overexpressing IGFBP3 lentivirus. (**E**) Immunoblotting assay of IGFBP3 expression in LN229 and T98G cells infected with lentivirus of targeting IGFBP3 (sh-IGFBP3) and control (sh-con). **(F)** Immunoblotting was performed to detect the expression of IGFBP3 in LN229 and T98G cells transfected with si-RNA targeting IGFBP3 (si-IGFBP3) and negative control (si-NC). Data are expressed as mean ± SD. **P<0.01; ***P<0.001, Student t test.
