# Supplementary material for: IGFBP3 induces PD-L1 expression to promote glioblastoma immune evasion
Source: Cancer Cell Int. 2024 Feb 7;24:60. doi: 10.1186/s12935-024-03234-3 (PMC10851611; doi:10.1186/s12935-024-03234-3)
Supplement: Supplementary file 4 — Additional file 4: Fig. S2. Immunoblotting was used to confirm that IGFBP3 regulates PD-L1 expression through the JAK2/STAT3 signaling pathway. [file 12935_2024_3234_MOESM4_ESM.docx]

**Supplementary figure 2**

**
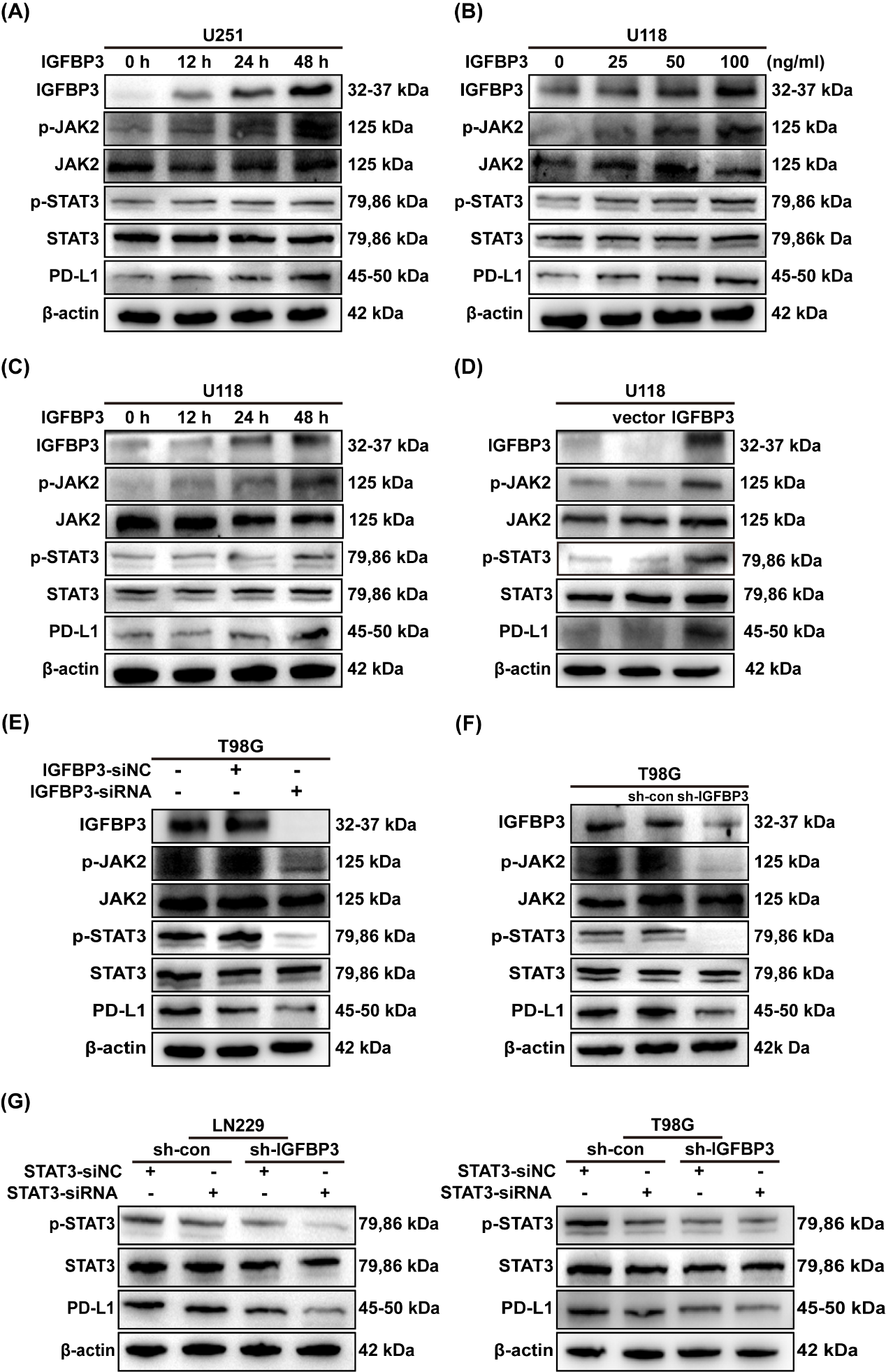
**

**Supplementary figure 2** Immunoblotting was used to confirm that IGFBP3 regulates PD-L1 expression through the JAK2/STAT3 signaling pathway. **(A)** Immunoblotting was performed to determine the expression of IGFBP3, p-JAK2, p-STAT3, and PD-L1 in U251 cell stimulated with 100 ng/mL of IGFBP3 at different points in time. **(B)** Immunoblotting analysis of IGFBP3, p-JAK2, p-STAT3, and PD-L1 expression in U118 cell treated with different concentrations of IGFBP3 for 24 hours. **(C)** Immunoblotting was performed to determine the expression of IGFBP3, p-JAK2, p-STAT3, and PD-L1 in U118 cell stimulated with 100 ng/mL of IGFBP3 at different points in time. **(D)** Immunoblotting analysis of IGFBP3, p-JAK2, p-STAT3, and PD-L1 expression in U118 cell infected with IGFBP3 overexpressing and empty vector lentivirus. **(E)** Immunoblotting was performed to examine the expression of IGFBP3, p-JAK2, p-STAT3, and PD-L1 in T98G cell transfected with si-RNA targeting IGFBP3 (si-IGFBP3) and negative control (si-NC). **(F)** Immunoblotting analysis of IGFBP3, p-JAK2, p-STAT3, and PD-L1 expression in T98G cell infected with sh-IGFBP3 and sh-con. **(G)** LN229 and T98G cells with IGFBP3 knockdown were transfected with si-RNA targeting STAT3 (si-STAT3) for 48 hours, cell lysate was used to analyze the expression of p-STAT3 and PD-L1.
