# Supplementary material for: IGFBP3 induces PD-L1 expression to promote glioblastoma immune evasion
Source: Cancer Cell Int. 2024 Feb 7;24:60. doi: 10.1186/s12935-024-03234-3 (PMC10851611; doi:10.1186/s12935-024-03234-3)
Supplement: Supplementary file 5 — Additional file 5: Fig. S3. Knockdown of IGFBP3 inhibits proliferation and invasion of LN229 and T98G cells. [file 12935_2024_3234_MOESM5_ESM.docx]

**Supplementary figure 3**

**
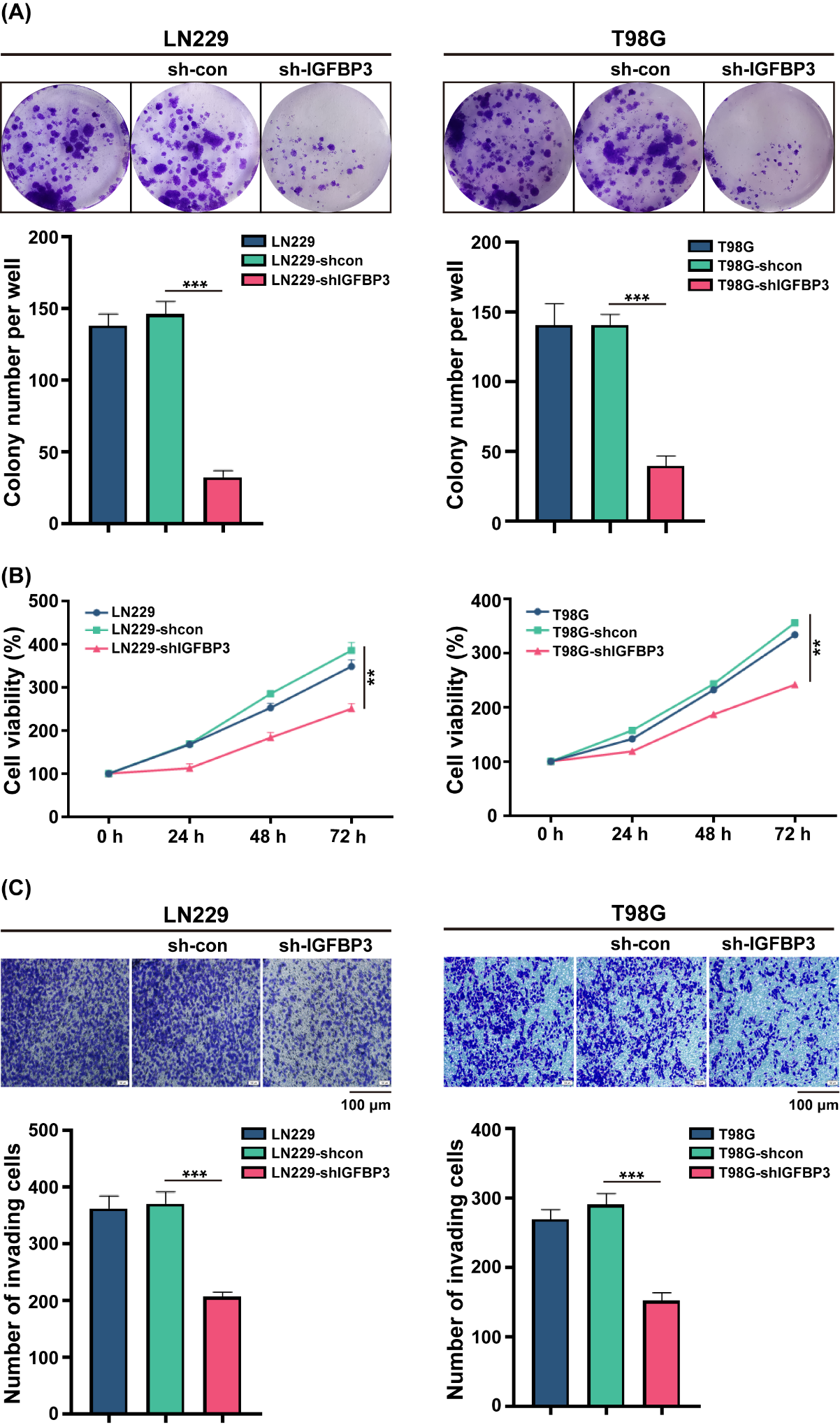
**

**Supplementary figure 3** Knockdown of IGFBP3 inhibits proliferation and invasion of LN229 and T98G cells. **(A)** Representative images of colony formation of LN229 and T98G cells infected with lentivirus of targeting IGFBP3 (sh-IGFBP3) and control (sh-con), Student t test. **(B)** The viability of LN229 and T98G cells infected with sh-IGFBP3 and sh-con was examined by MTT assay, Student t test. **(C)** Transwell assay of LN229 and T98G cells infected with sh-IGFBP3 and sh-con, Student t test. Data are expressed as mean ± SD. **P<0.01; ***P<0.001, Student t test.
